# Xanthogranulomatous Osteomyelitis as a Bone Tumor Mimic: A Systematic Review of Pre-biopsy Diagnostic Clues and Management Decision Points

**DOI:** 10.7759/cureus.109978

**Published:** 2026-05-31

**Authors:** Ethan J Sack, Max Deneroff, Austin Khau, Zubair Mohammed, Chadi Ellaham, Jared W Nichols

**Affiliations:** 1 College of Osteopathic Medicine, Kansas City University, Joplin, USA; 2 Osteopathic Manipulative Medicine, Kansas City University, Joplin, USA

**Keywords:** bone tumor mimic, chronic osteomyelitis, foamy macrophages, integrated diagnostic framework, lytic bone lesion, xanthogranulomatous osteomyelitis

## Abstract

Xanthogranulomatous osteomyelitis (XO) is a rare inflammatory form of osteomyelitis that can closely resemble a primary bone tumor or metastatic lesion at presentation. This systematic review was conducted in accordance with Preferred Reporting Items for Systematic Reviews and Meta-Analyses (PRISMA) guidelines and focused on pre-biopsy diagnostic clues and management decision points in published skeletal XO reports. PubMed, Embase, and Scopus were searched from January 2026 to May 2026, identifying 86 records. Eligible studies included human case reports and case series describing histologically confirmed skeletal XO or xanthogranulomatous inflammation involving bone; non-skeletal xanthogranulomatous disease, duplicate reports, review articles without extractable patient-level data, and distinct neoplastic entities were excluded. After deduplication and full-text review, 31 studies met the inclusion criteria and represented 33 patients. Data extraction was performed independently by two reviewers, and reporting quality and risk of bias were assessed using the Joanna Briggs Institute critical appraisal checklist for case reports. A neoplastic process was considered before biopsy in 29 of 31 studies (93.5%). Lytic or osteolytic destruction was described in 25/31 (80.6%), periosteal reaction in 20/31 (64.5%), a soft-tissue component in 19/31 (61.3%), and cortical breach or destruction in 16/31 (51.6%). Biopsy or histopathologic confirmation was reported in 22/31 studies (71.0%), and organism or culture data were documented in 19/31 (61.3%), supporting combined histopathologic and microbiologic evaluation. Management ranged from biopsy and debridement to reconstructive or resection procedures, depending on diagnostic uncertainty and structural risk. Overall, the literature supports XO as an important skeletal tumor mimic. An integrated clinical-radiologic-pathologic framework with early tissue confirmation and routine culture submission when tissue is obtained may help avoid inappropriate oncologic treatment and guide lesion-specific management.

## Introduction and background

Xanthogranulomatous osteomyelitis (XO) is an uncommon chronic inflammatory disorder of bone characterized histologically by lipid-laden foamy macrophages within a mixed inflammatory background that may include plasma cells, lymphocytes, multinucleated giant cells, and necrotic bone [1]. Xanthogranulomatous inflammation is more often reported in organs such as the kidney or gallbladder, making skeletal involvement an unusual diagnostic consideration [1-3]. Since the earliest descriptions of XO, the skeletal literature has remained limited to isolated case reports and small case series, limiting the ability to define a consistent clinical phenotype or standardized treatment pathway [4-34].

The clinical relevance of XO lies in its ability to imitate aggressive infectious or neoplastic bone disease. Patients may present with localized pain, swelling, tenderness, or a palpable mass, while fever and elevated inflammatory markers are inconsistently reported. Imaging can further complicate the diagnosis because reported cases have described lytic or osteolytic lesions, expansile osseous destruction, cortical breach, periosteal reaction, marrow edema, soft-tissue extension, and increased fluorodeoxyglucose (FDG) uptake on positron emission tomography/computed tomography (PET/CT). These findings overlap with pyogenic osteomyelitis, tuberculous osteomyelitis, osteosarcoma, Ewing sarcoma, metastasis, multiple myeloma, Langerhans cell histiocytosis, Erdheim-Chester disease, and abscess [2,3,8-13,18,20,32,33]. As a result, several reported cases underwent referral or evaluation for suspected malignancy before tissue diagnosis.

Histopathologic evaluation remains central to diagnosis. Demonstration of foamy histiocytes in an appropriate inflammatory and osseous context helps separate XO from malignant neoplasms and other histiocytic disorders [1]. Microbiologic testing is also important because an infectious driver may be identified in selected cases, although culture results are inconsistently reported and range from culture-negative disease to bacterial or fungal organisms. Bone biopsy with microbial culture is widely recognized as a definitive diagnostic approach in osteomyelitis evaluation [2]. Together, these findings suggest that XO may represent a final inflammatory reaction pattern rather than a single uniform infectious entity, supporting combined histopathologic and microbiologic assessment whenever tissue is obtained [2,4,10-13,17,30].

Management is similarly variable. The reported management approaches ranged from diagnostic biopsy and antimicrobial therapy to curettage, debridement, bone grafting, fixation, arthroplasty, vertebrectomy, and resection. In practice, treatment appears to depend on whether infection is identified, whether the lesion threatens mechanical stability or neurologic structures, and whether malignancy can be confidently excluded. A clinically useful synthesis should therefore clarify not only reported cases of XO but also the pre-biopsy findings that raise concern for malignancy and the post-biopsy findings that guide treatment selection [12-14,17,22,30,31].

Rather than providing a broad catalog of reported XO cases, this review was organized around a practical diagnostic question: among reported cases of skeletal XO, which clinical and imaging features led clinicians to consider malignancy before biopsy, and how did histopathology and culture findings influence subsequent management? We therefore focus on XO as a skeletal tumor mimic and evaluate the clinical, laboratory, and imaging findings that most often raise concern for malignancy before biopsy. Based on these findings, we propose an integrated clinical-radiologic-pathologic diagnostic framework with early tissue confirmation that incorporates histopathology, culture results, anatomic site, and structural risk.

## Review

Methods

Study Design

We conducted a descriptive systematic review of published skeletal XO case reports and small case series in accordance with Preferred Reporting Items for Systematic Reviews and Meta-Analyses (PRISMA) guidelines [35].

Search Strategy and Data Sources

A structured search was performed from January 2026 to May 2026 in PubMed, Embase, and Scopus only. The review was reported in accordance with PRISMA 2020 guidance [35]. Searches used exact-phrase and free-text terms for XO, and record counts were documented before duplicate removal. Database-specific search strings and raw record counts are provided in Table 1. The combined search yielded 86 records: PubMed (n=29), Embase (n=23), and Scopus (n=34).

**Table 1:** Database-specific search strategies and identified records

Database	Search strategy	Records identified
PubMed	("xanthogranulomatous osteomyelitis"[Title/Abstract] OR ("xanthogranulomatous"[Title/Abstract] AND "osteomyelitis"[Title/Abstract]))	29
Embase	('xanthogranulomatous osteomyelitis':ti,ab,kw OR (xanthogranulomatous:ti,ab,kw AND osteomyelitis:ti,ab,kw))	23
Scopus	TITLE-ABS-KEY("xanthogranulomatous osteomyelitis" OR (xanthogranulomatous AND osteomyelitis))	34
Total	PubMed + Embase + Scopus	86

Study Eligibility and Selection

Eligible publications included human case reports, small case series, and other case-based reports describing histologically confirmed skeletal XO or xanthogranulomatous inflammation involving bone. Because XO is rare, case-based evidence was considered appropriate for descriptive synthesis. Reports were excluded if they described non-skeletal xanthogranulomatous disease, lacked extractable patient-level data, represented duplicate patient reports, were review-only articles without new patient-level information, had insufficient pathologic confirmation of skeletal XO, or described distinct neoplastic entities rather than conventional XO. Review articles without new patient-level data were used only for reference-list checking and were not treated as independent cases.

Titles and abstracts were screened by two reviewers. Potentially eligible reports then underwent independent full-text review by two reviewers. Disagreements regarding eligibility or extracted data were resolved by discussion and consensus. Final exclusion reasons were documented and incorporated into the PRISMA flow diagram.

Outcome Measures

The primary outcomes were pre-biopsy tumor-mimic features, tissue diagnosis, procedure type, culture results, antimicrobial use, clinical resolution, recurrence, follow-up duration, and complications. Secondary outcomes included anatomic distribution, symptoms, inflammatory markers, and the working diagnosis before biopsy.

Data Extraction and Synthesis

Data were extracted independently by two reviewers using a standardized data extraction form. Extracted variables included publication year, country, study type, patient age and sex, involved bone and lesion location, presenting symptoms, inflammatory markers when reported, imaging features, pre-biopsy working diagnosis, histopathologic findings, microbiology and culture results, antimicrobial or antifungal therapy, procedure type, clinical outcome, recurrence, follow-up duration, and complications. Disagreements in extracted variables were resolved through discussion and consensus.

Because the included evidence consisted primarily of case reports and small case series without comparator groups, standardized outcome definitions, or uniform follow-up intervals, meta-analysis and meta-regression were not feasible. Findings were therefore synthesized narratively and summarized using descriptive statistics. Study-level proportions were calculated using the 31 included studies as the denominator, while patient-level demographic summaries were calculated using the 33 represented patients as the denominator. Percentages were used only to describe patterns within the included case literature and were not interpreted as incidence estimates or comparative effect measures.

For key study-level proportions, 95% confidence intervals were calculated using the Wilson method to reflect uncertainty associated with small denominators. Key proportions included pre-biopsy consideration of malignancy, lytic or osteolytic imaging, cortical breach or destruction, periosteal reaction, soft-tissue component, biopsy or histopathologic confirmation, culture or organism reporting, and favorable clinical or radiographic outcome. The synthesis was organized around the clinical sequence of presentation, imaging, pre-biopsy differential diagnosis, tissue diagnosis, microbiology, management, and follow-up.

Assessment of Bias

Case reports and case series were appraised using the Joanna Briggs Institute (JBI) Critical Appraisal Checklist for Case Reports (Table 2) [36]. Appraisal was performed independently by two reviewers, and disagreements were resolved by discussion and consensus. Each report was assessed across the standard JBI domains, including demographic clarity, patient history and timeline, presenting clinical condition, diagnostic methods and results, intervention or treatment description, post-intervention clinical status, adverse events or harms, and takeaway lessons. The appraisal results were used to contextualize completeness of reporting and risk of bias, not to exclude otherwise eligible reports.

**Table 2:** JBI critical appraisal summary Q1: demographics described; Q2: history/timeline described; Q3: clinical condition described; Q4: diagnostic methods/results described; Q5: interventions described; Q6: post-intervention condition described; Q7: adverse events/harms described; Q8: takeaway lessons provided JBI, Joanna Briggs Institute

Studies	Q1	Q2	Q3	Q4	Q5	Q6	Q7	Q8
Solooki et al. (2019) [4]	Y	Y	Y	Y	Y	Y	Y	N
Borjian et al. (2012) [5]	Y	Y	Y	Y	Y	Y	Y	N
Kamat et al. (2011) [6]	Y	Y	Y	Y	Y	Y	Y	N
Cozzutto (1984) [7]	Y	Y	Y	Y	Y	Y	Y	N
Kaneuchi et al. (2017) [8]	Y	Y	Y	Y	Y	Y	Y	N
Mohanan et al. (2025) [9]	Y	Y	Y	Y	Y	Y	Y	N
Wang et al. (2014) [10]	Y	Y	Y	Y	N	N	N	N
Vankalakunti et al. (2007) [11]	Y	Y	Y	Y	Y	Y	Y	N
Lee and Moon (2024) [12]	Y	Y	Y	Y	Y	Y	Y	N
Perera et al. (2024) [13]	Y	Y	Y	Y	Y	Y	Y	N
Cheema et al. (2017) [14]	Y	Y	Y	Y	Y	Y	Y	N
Scicluna et al. (2020) [15]	Y	Y	Y	Y	Y	Y	Y	N
Gupta et al. (2025) [16]	Y	Y	Y	Y	Y	Y	N	Y
Choi et al. (2024) [17]	Y	Y	Y	Y	Y	Y	Y	N
Yadav et al. (2023) [18]	Y	Y	Y	Y	Y	Y	Y	N
Ju et al. (2023) [19]	Y	Y	Y	Y	Y	Y	Y	N
Manjula et al. (2021) [20]	Y	Y	Y	Y	Y	Y	N	N
Bloch-Maier et al. (2025) [21]	Y	Y	Y	Y	Y	Y	Y	N
Murthy et al. (2025) [22]	Y	Y	Y	Y	Y	Y	Y	N
Bali et al. (2019) [23]	Y	Y	Y	Y	Y	Y	N	N
Singh et al. (2015) [24]	Y	Y	Y	Y	Y	Y	Y	N
Hatakeyama et al. (2025) [25]	Y	Y	Y	Y	Y	Y	Y	N
Sapra et al. (2015) [26]	Y	Y	Y	Y	Y	Y	Y	N
Rathi et al. (2014) [27]	Y	Y	Y	Y	Y	Y	N	N
Hota et al. (2022) [28]	Y	Y	Y	Y	Y	Y	N	N
Bae et al. (2023) [29]	Y	Y	Y	Y	Y	Y	Y	N
Pathak et al. (2019) [30]	Y	Y	Y	Y	Y	Y	Y	N
Nair et al. (2025) [31]	Y	Y	Y	Y	Y	Y	Y	N
Arul et al. (2016) [32]	Y	Y	Y	Y	Y	Y	N	N
Lee et al. (2013) [33]	Y	Y	Y	Y	Y	Y	Y	N
Singhal and Shekhar (2020) [34]	Y	Y	Y	Y	Y	Y	N	N

Results

Study Selection

**Figure 1:**
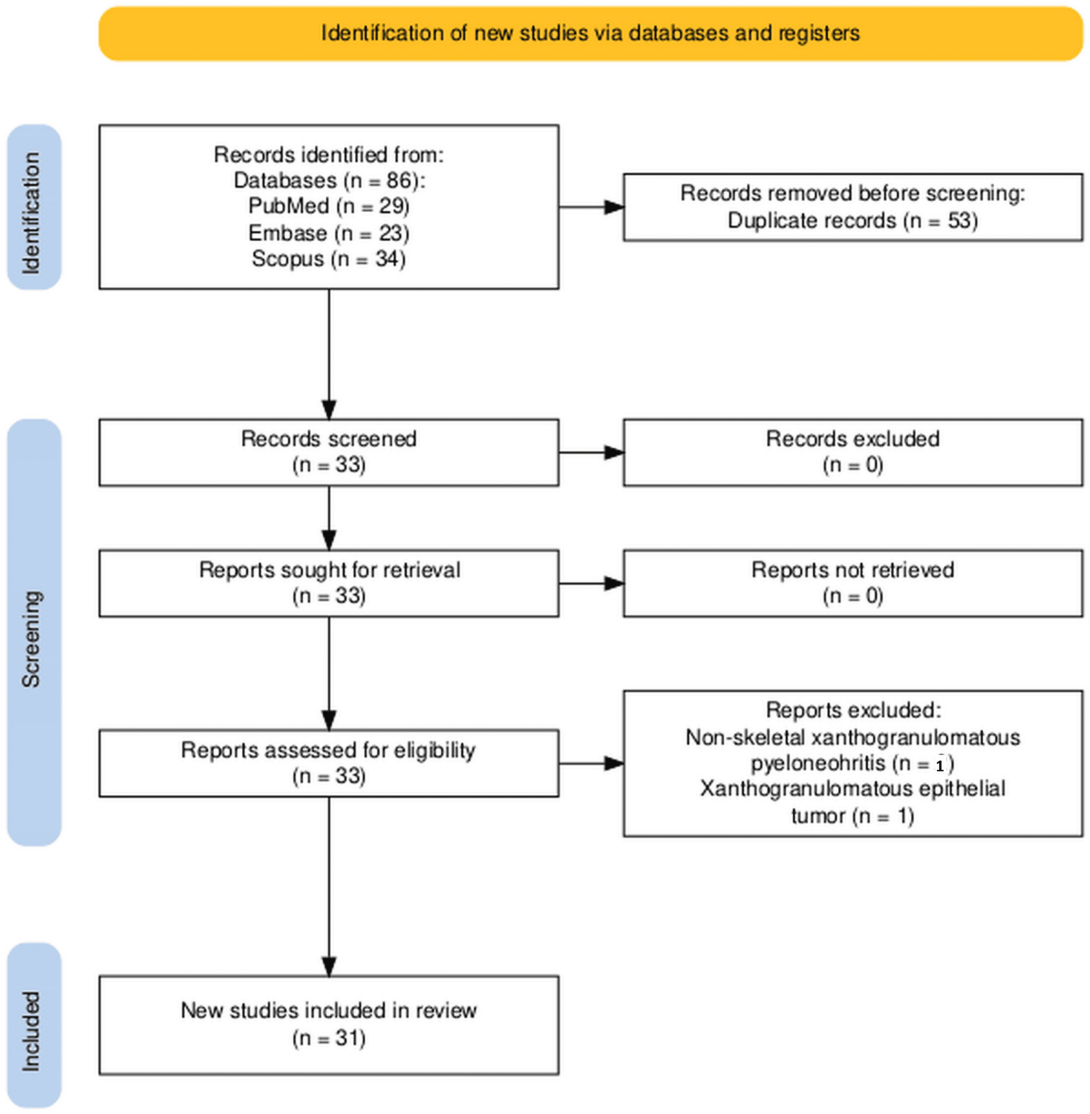
PRISMA flow diagram Flow of records through the systematic review process. Database searches included PubMed, Embase, and Scopus. After the removal of 53 duplicate records, 33 records were screened and underwent full-text assessment. Two full-text reports were excluded: one non-skeletal xanthogranulomatous pyelonephritis report and one xanthogranulomatous epithelial tumor report. Thirty-one studies were included in the qualitative synthesis. The diagram was prepared in a PRISMA 2020-compatible format using the PRISMA2020 Shiny/R flow diagram tool [37]. PRISMA, Preferred Reporting Items for Systematic Reviews and Meta-Analyses

Database searches of PubMed, Embase, and Scopus identified 86 records, of which 53 were duplicates. The remaining 33 records underwent title and abstract screening; no records were excluded at that stage, as all were potentially relevant case-based literature requiring full-text review. All 33 full-text reports were retrieved and assessed for eligibility. Two reports were excluded from the final synthesis: one described non-skeletal xanthogranulomatous pyelonephritis, and one described a xanthogranulomatous epithelial tumor, which represents a distinct neoplastic entity rather than conventional XO. Thirty-one studies were included in the qualitative synthesis. The study selection process is summarized in Figure 1.

Data Extraction Summary

Study characteristics and extracted patient-level variables are summarized in Table 3. The table emphasizes features relevant to the tumor-mimic question, including pre-biopsy diagnostic concern, imaging aggressiveness, microbiology, treatment approach, and outcomes.

**Table 3:** Condensed data extraction summary of included XO case reports and case series XO, xanthogranulomatous osteomyelitis; MRI, magnetic resonance imaging; TB, tuberculosis; CT, computed tomography; 18FDG PET/CT, fluorodeoxyglucose positron emission tomography/computed tomography; ROM, range of motion

Study/report	Year	Age/sex	Bone/site	Pre-biopsy concern	Management	Outcome
Solooki et al., 2019 [4]	2019	15/M	Right tibia, proximal metaphysis	Osteomyelitis/abscess vs. Ewing sarcoma vs. osteosarcoma; tumor mimic workup	Biopsy; antibiotics reported; no curettage in the original case report	Symptoms resolved; no radiographic recurrence at 11 weeks; inflammatory markers normalized later
Borjian et al., 2012 [5]	2012	14/M	Right humerus and left fibula	Multifocal tumor/infection mimic	Antibiotics after diagnosis; no major surgery reported as primary management	Resolution/no recurrence
Kamat et al., 2011 [6]	2011	13/M	Right tibia	Bone tumor/chronic osteomyelitis; abscess/infectious differential	Simple curettage	Curative/resolution
Cozzutto, 1984 [7]	1984	5/M and 14/M	First rib; tibia	Chronic osteomyelitis/tumor-like inflammatory lesions	Surgery plus antibiotics	Resolution/no recurrence in prior synthesis
Kaneuchi et al., 2015/2017 [8]	2015/2017	36/F	Distal tibia	Tumor-like distal tibial lesion; Langerhans/histiocytic and infection are considered	Biopsy with excision or curettage as described in the report	Resolution/no recurrence reported in the review table
Mohanan et al., 2025 [9]	2025	42/F	Left tibial diaphysis	Ewing sarcoma, metastasis/sarcoma, and histiocytic disease are considered	Biopsy, curettage, and grafting	Healing/no recurrence or favorable follow-up reported
Wang et al., 2014 [10]	2014	45/M and 46/M	Ribs: left 3rd rib; right 8th rib and left 5th rib	Malignant lesions on 18F-FDG PET/CT	Case 1 underwent resection; case 2 had biopsy/histopathologic confirmation, and management details were limited in the article	Case 1 resolution; case 2 outcome not fully reported
Vankalakunti et al., 2007 [11]	2007	50/F	Right ulna diaphysis	Neoplasm/malignancy mimic	Curettage specimen/biopsy; surgical management	No mortality; lost to follow-up in prior synthesis
Lee and Moon, 2024 [12]	2024	23/F	Right pubic bone/pubic tubercle	Cystic bone tumor vs. TB osteomyelitis vs. acute osteomyelitis/abscess	Excisional biopsy, curettage, and irrigation; antifungal therapy after Aspergillus identification	No recurrence through follow-up
Perera et al., 2024 [13]	2022 online/2024 issue	65/F	C5-C7 cervical spine	Metastatic cord compression/malignancy; abscess/sarcoma/histiocytic disorders considered	C5-C7 vertebrectomy with cage/anterior plating; no antibiotics reported	Neurologic improvement; no recurrence at 2 years
Cheema et al., 2017 [14]	2017	5/F	Right humerus	Ewing sarcoma/sarcoma vs. infection; Langerhans cell histiocytosis considered	Open biopsy/debridement plus antibiotics	Resolution reported in prior synthesis
Scicluna et al., 2020 [15]	2020	20/M	Right pubic bone/superior pubic ramus	Crohn-related inflammatory/infectious lesion; TB excluded	CT-guided biopsy; immunosuppressive therapy; no surgery	Resolution/no recurrence reported
Gupta et al., 2025 [16]	2025	25/M	Left forearm bone (specific bone not specified in the extracted report)	Osteomyelitis vs. neoplasm/malignancy mimic	Histopathologic evaluation; treatment not specified in the extracted data	Not reported
Choi et al., 2024 [17]	2024	41/M	Sternoclavicular region: clavicle/sternum/manubrium	Malignancy/sarcoma concern; initial biopsy suggested inflammatory myofibroblastic tumor	Complete resection with partial clavicle/sternum removal	No recurrence reported
Yadav et al., 2023 [18]	2023	64/M	Temporal bone	Malignancy/temporal bone tumor mimic; inflammatory/infectious differential	Biopsy/mastoid surgery plus antibiotics	Clinical improvement/stable at short follow-up
Ju et al., 2023 [19]	2023	15/F	Distal fibula/lateral malleolar region	Subacute infection vs. Ewing/sarcoma/abscess	Curettage/debridement	Improved/healing
Jayanti/Manjula et al., 2021 [20]	2021	22/F	Left fourth finger/phalangeal region	Enchondroma/neoplasm on MRI	Curettage with histopathologic confirmation	Healing/improvement
Bloch-Maier et al., 2025 [21]	2025	16/M	Left mandible/mandibular angle	Fibrous dysplasia, desmoplastic fibroma, ameloblastoma, keratocyst, Ewing sarcoma, osteosarcoma	Incisional biopsy followed by observation/monitoring	Radiographic regression/healing by follow-up
Murthy et al., 2025 [22]	2025	24/M	Left proximal femur/hip	Infective etiology, pyogenic or tubercular; tumor mimic context	Two-stage procedure: excision arthroplasty/spacer followed by total hip arthroplasty	Favorable clinical outcome reported
Bali et al., 2019 [23]	2019	80/F	Left intertrochanteric/proximal femur	Fracture/infection; malignancy excluded histologically	Treatment not specified in the extracted data	Not reported
Singh et al., 2015 [24]	2015	65/F	Right proximal femur/peritrochanteric region	Benign bone tumor, fibrous dysplasia, Langerhans/Erdheim-Chester; malignancy considered	Biopsy, curettage, bone grafting, and fixation as reported	Healed/resolution
Hatakeyama et al., 2025 [25]	2025	50/M	Right femur/lower metaphyseal region	Primary bone tumor; malignant bone tumor mimic	Biopsy; antibiotics/medical treatment response described	Favorable clinical/lab response without adverse events
Sapra et al., 2015 [26]	2015	34/M	Multifocal bilateral tarsal/foot bones	Tuberculosis/metastasis/multifocal osteomyelitis is considered	Biopsy, curettage, resection, and fixation as described in the report	Improved/favorable outcome; recurrence status was not clearly reported
Rathi et al., 2014 [27]	2014	50/M	Left distal tibia/ankle region	TB/chronic osteomyelitis/tumor mimic	Excisional biopsy, curettage, and grafting	Not reported
Hota et al., 2022 [28]	2022	43/M	Tibia	Lytic bone lesion; differential included tumor/TB/osteomyelitis	Biopsy; antibiotic regimen pending culture	Not reported
Bae et al., 2023 [29]	2023	81/F	Right mandibular body/lower jaw	Jaw lesion; malignancy/infection considered as a clinically relevant differential	Incisional biopsy; surgical management/grafting as reported	Resolved/improved/healed
Pathak et al., 2019 [30]	2019	50/F	Left hip: femoral head/acetabulum/ilium	Tubercular osteomyelitis/septic arthritis; benign ilium lesion	Debridement, girdlestone resection arthroplasty, and curettage; antibiotics	Clinical/lab improvement; residual functional issues at follow-up
Nair et al., 2025 [31]	2025	62/M	Right radius mid-shaft	Osteoblastoma vs. multiple myeloma	Excision biopsy, plate fixation, and fibular grafting	No recurrence; healed with full ROM at 2 years
Arul et al., 2016 [32]	2016	20/M	Lower metaphyseal femur	Primary bone tumor/malignant bone tumor mimic	Histopathologic evaluation; treatment not specified in the extracted data	Not reported
Lee et al., 2013 [33]	2013	59/M	Distal ulna	Malignancy/metastasis/myeloma/histiocytic lesion considered	Excisional biopsy/lesion excision	Stable/no recurrence reported
Singhal and Shekhar, 2020 [34]	2020	26/M	Left humerus/arm	Chronic osteomyelitis vs. malignancy mimic	Needle biopsy/biopsy and debridement	Not reported

Study Characteristics and Demographics

After manual verification, 31 studies representing 33 patients were included. Patient age was reported in all included studies, with a median of 36 years (range, 5-81 years). At the patient level, 21/33 patients (63.6%) were male and 12/33 (36.4%) were female. Reported cases spanned pediatric, adolescent, adult, and elderly patients, suggesting that XO is not restricted to a narrow demographic profile. Several reports described antecedent trauma, chronic infection, or immunosuppression, although many involved otherwise immunocompetent patients [12,14-17].

Anatomic Distribution

**Figure 2:**
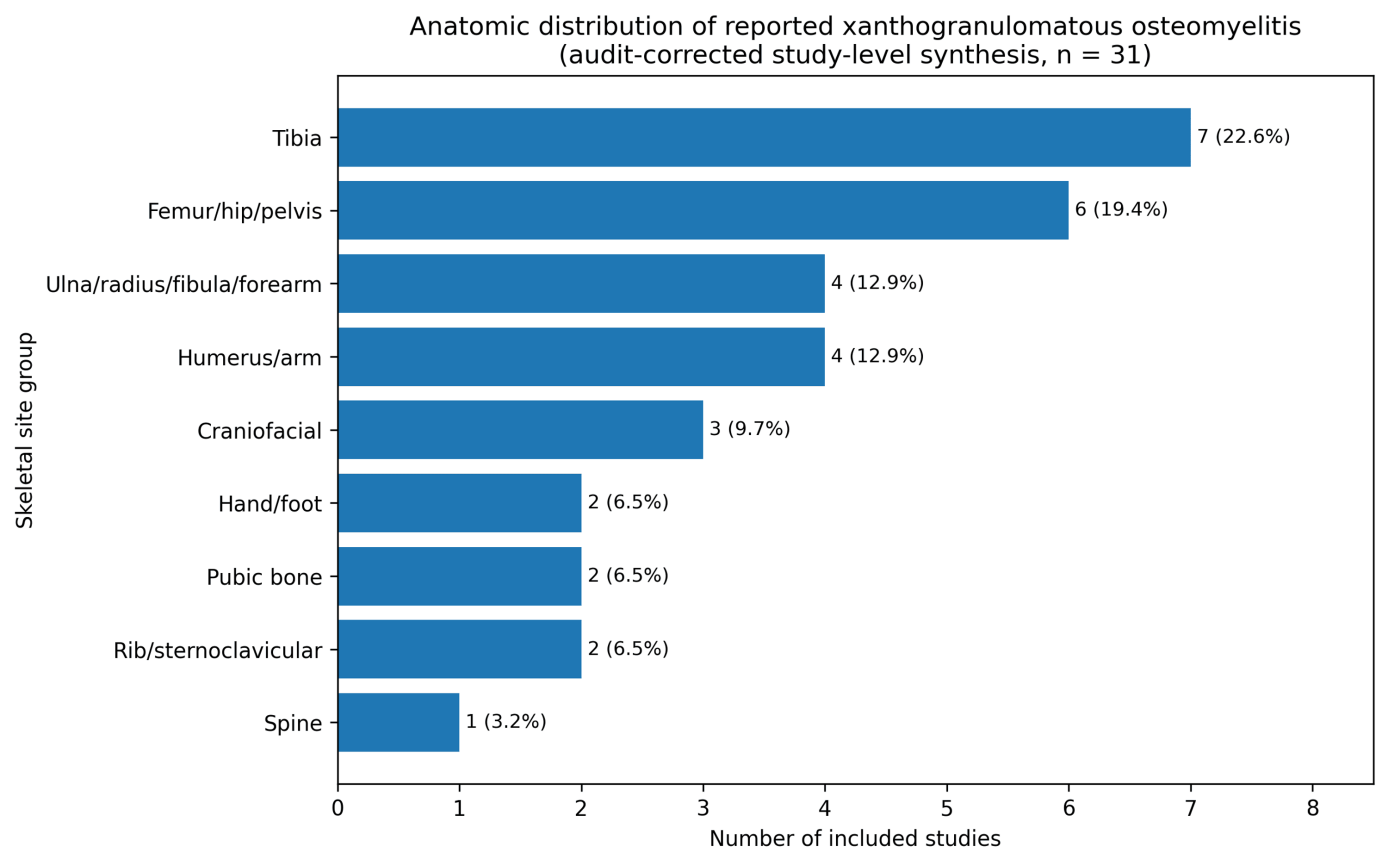
Anatomic distribution of reported XO by skeletal site group Bars display the number and percentage of the 31 included studies within each skeletal site group. XO, xanthogranulomatous osteomyelitis

Reported skeletal involvement was heterogeneous. The tibia was the most frequently described site group (7/31, 22.6%), followed by femur/hip/pelvis involvement (6/31, 19.4%). Humerus/arm and ulna/radius/fibula/forearm involvement each accounted for 4/31 (12.9%) and 4/31 (12.9%), respectively. Craniofacial involvement was also represented, including temporal bone and mandibular cases. This broad anatomic distribution reinforces the notion that XO can arise in both appendicular and axial skeletal sites and may present in regions where destructive lesions commonly prompt concern for malignancy (Figure 2).

Clinical Presentation

Pain remained the dominant presenting symptom after denominator correction (26/31, 83.9%), followed by swelling (24/31, 77.4%), mass effect (23/31, 74.2%), tenderness (20/31, 64.5%), and fever (18/31, 58.1%). These features were nonspecific and overlapped substantially with osteomyelitis, malignancy, and chronic inflammatory lesions. Accordingly, clinical presentation alone rarely distinguished XO from sarcoma, metastatic disease, tuberculosis, abscess, or other histiocytic disorders [4,12,13,30].

Imaging Findings and Pre-biopsy Differential Diagnosis

Advanced cross-sectional imaging was reported in 29 of 31 studies (93.5%). Lytic or osteolytic destruction was described in 25/31 reports (80.6%), periosteal reaction in 20/31 (64.5%), a soft-tissue component in 19/31 (61.3%), and cortical breach or destruction in 16/31 (51.6%). A neoplastic or malignant process was included in the pre-biopsy differential in 29/31 studies (93.5%), while tuberculosis was considered in 7/31 (22.6%). These findings support the role of XO as a skeletal tumor mimic and highlight why imaging should guide lesion characterization and biopsy planning rather than serve as the sole basis for definitive treatment [4-7,11].

Histopathology, Culture, and Management

Management decisions were closely linked to tissue diagnosis. Histopathologic confirmation of XO helped redirect care away from primary oncologic treatment in cases initially concerning for malignancy and supported less aggressive management when structural risk was limited. In several reports, biopsy or excision established the diagnosis and was followed by curettage, debridement, grafting, fixation, or observation depending on lesion location and mechanical stability. Culture results further influenced management when an organism was identified. Culture-positive cases prompted organism-directed antimicrobial or antifungal therapy, including cases in which bacterial or fungal pathogens were reported, whereas culture-negative cases were generally managed according to histopathologic diagnosis, lesion burden, and structural risk rather than prolonged empiric antimicrobial therapy alone. Overall, curettage, debridement, or irrigation was described in 14/31 studies (45.2%), while resection, excision, arthroplasty, fixation, grafting, or other reconstructive procedures were described in 14/31 (45.2%). Antimicrobial, antifungal, or medical therapy was described in 10/31 studies (32.3%). Thus, histopathology primarily determined whether the lesion should be treated as inflammatory rather than malignant, while culture results determined whether targeted antimicrobial therapy was required. Complex lesions involving the spine, pelvis, hip, mandible, temporal bone, or sternoclavicular region required site-specific surgical decisions, particularly when neurologic compromise, fracture risk, joint involvement, or extensive cortical destruction was present [12,13,17,18].

Clinical Outcomes

Outcome reporting was heterogeneous. Favorable clinical or radiographic outcomes were reported in 22/31 studies (71.0%), while 7/31 (22.6%) had incompletely reported outcomes. The overall pattern suggests that once XO is recognized and managed based on structural risk and microbiologic findings, clinical improvement is common. However, inconsistent reporting of antimicrobial duration, functional recovery, and recurrence limits strong inference.

*Quantitative Summary*

Key study-level clinical, imaging, diagnostic, management, and outcome findings are summarized in Table 4 with descriptive percentages and 95% confidence intervals.

**Table 4:** Quantitative synthesis of key clinical, imaging, diagnostic, management, and outcome findings Frequencies are reported at the study level using the 31 included studies as the denominator. Percentages are descriptive and should not be interpreted as incidence estimates or comparative effect measures. Confidence intervals were calculated using the Wilson method. Management categories are not mutually exclusive because some reports described combined biopsy, debridement, reconstruction, and antimicrobial therapy.

Domain	Variable	Frequency	Percentage	95% CI
Presentation	Pain reported	26/31	83.90%	67.4-92.9%
	Swelling reported	24/31	77.40%	60.2-88.6%
	Mass effect reported	23/31	74.20%	56.8-86.3%
	Tenderness reported	20/31	64.50%	46.9-78.9%
	Fever reported	18/31	58.10%	40.7-73.6%
Imaging	Lytic/osteolytic lesion	25/31	80.60%	63.7-90.8%
	Expansile lesion	07/31	22.60%	11.4-39.8%
	Cortical breach/destruction	16/31	51.60%	34.8-68.0%
	Periosteal reaction	20/31	64.50%	46.9-78.9%
	Soft-tissue component	19/31	61.30%	43.8-76.3%
	Advanced cross-sectional imaging reported	29/31	93.50%	79.3-98.2%
Pre-biopsy differential	Neoplasm/malignancy considered	29/31	93.50%	79.3-98.2%
	Tuberculosis considered	07/31	22.60%	11.4-39.8%
	Infection/abscess/chronic osteomyelitis considered	20/31	64.50%	46.9-78.9%
Microbiology	Any organism/culture result reported	19/31	61.30%	43.8-76.3%
	Staphylococcus reported among included studies	06/31	19.40%	9.2-36.3%
Management	Biopsy/histopathology reported	22/31	71.00%	53.4-83.9%
	Curettage/debridement/irrigation reported	14/31	45.20%	29.1-62.2%
	Resection/excision/reconstruction reported	14/31	45.20%	29.1-62.2%
	Antimicrobial, antifungal, or medical therapy described	10/31	32.30%	18.6-49.9%
Outcomes	Favorable clinical/radiographic outcome reported	22/31	71.00%	53.4-83.9%
	Outcome unclear or incompletely reported	07/31	22.60%	11.4-39.8%
	Explicit follow-up duration extracted	05/31	16.10%	7.1-32.6%

Reporting Quality

The JBI appraisal showed that most included reports provided adequate demographic information, clinical presentation, diagnostic evaluation, and treatment descriptions. The most frequent reporting limitations were incomplete longitudinal follow-up, inconsistent adverse-event reporting, and variable detail regarding microbiology, antimicrobial duration, and functional outcomes. Because the included evidence consisted primarily of case reports and small case series, the appraisal results were used to contextualize completeness of reporting and risk of bias.

Discussion

This review was intentionally structured around XO as a tumor-mimicking skeletal lesion rather than as a general catalog of published cases. A neoplastic or malignant process was included in the pre-biopsy differential diagnosis in 29/31 studies (93.5%), and at least half of the included studies reported cortical involvement, periosteal reaction, or a soft-tissue component. These findings help explain why XO may be mistaken for sarcoma, metastasis, or other aggressive bone pathology and support the value of an integrated diagnostic framework with early tissue confirmation.

The anatomic distribution of XO further contributes to diagnostic uncertainty. Although long bones such as the tibia and femur were common, reported cases also involved the ulna, radius, fibula, ribs, pubic bone, sternoclavicular region, temporal bone, mandible, jaw, and cervical spine [4,8-13,17-21,24,29-31]. This wide range of skeletal sites is clinically important because the suspected diagnosis often depends on where the lesion occurs: destructive lesions in long bones may raise concern for Ewing sarcoma or osteosarcoma, rib or sternoclavicular lesions may suggest metastasis or chest-wall malignancy, craniofacial lesions may mimic odontogenic or skull-base tumors, and cervical spine lesions may be approached initially as metastatic cord compression [10,13,17,18,21,24,29].

Radiographic and advanced imaging findings were central to the tumor-mimic problem. Several reports described destructive osteolysis, cortical compromise, periosteal reaction, marrow signal abnormality, extraosseous or soft-tissue extension, or FDG avidity, none of which is specific for XO [8-13,19,24,25,32,33]. In particular, FDG PET/CT avidity and enhancing soft-tissue components can amplify concern for malignancy even when infection or chronic inflammation remains possible [10,12,33]. Therefore, imaging should be interpreted to define lesion extent, biopsy trajectory, structural risk, and surgical planning rather than as a definitive discriminator between XO and malignancy.

Histopathologic confirmation remains the diagnostic pivot of XO. Across the case literature, the recurring pathologic pattern is a xanthogranulomatous inflammatory infiltrate composed of foamy histiocytes and macrophages, with variable numbers of lymphocytes, plasma cells, and giant cells. It may contain necrotic bone or chronic inflammatory debris [4-7,11,13,21,29]. This histologic pattern is important because it redirects management away from primary oncologic treatment while still requiring exclusion of mimics such as Langerhans cell histiocytosis, Erdheim-Chester disease, chronic granulomatous infection, plasma cell neoplasm, and rare xanthogranulomatous tumor entities [11,13,21,29]. The distinction is not merely semantic: misclassification could lead to either unnecessary wide resection or undertreatment of infection or structural instability.

Microbiologic data were less consistently reported than histopathology. Some cases identified organisms such as Staphylococcus aureus, Salmonella, Aspergillus, or other infectious agents. In contrast, others were culture-negative or did not clearly separate tissue culture from wound culture or prior antibiotic exposure [4,12,14,17,19,26,30]. This variability suggests that XO may not represent a single microbiologic disease. Instead, it may be best understood as a histologic reaction pattern that can occur in the setting of infection, chronic inflammation, immune dysregulation, or local tissue breakdown. For that reason, biopsy specimens should ideally be submitted for both histopathology and bacterial, fungal, and mycobacterial studies when clinically appropriate [4,12,17,30].

Management decisions were highly dependent on anatomic site and mechanical risk. Contained long-bone lesions were commonly managed with biopsy, curettage, debridement, or grafting, whereas lesions with pathologic fracture or major cortical compromise required fixation, grafting, arthroplasty, or reconstruction [8,9,22,24,25,31,32]. Anatomically complex disease required more individualized treatment: cervical spine involvement required decompression and reconstruction, temporal bone disease required skull-base/otologic management, sternoclavicular disease was treated with resection in a transplant recipient, pubic bone disease required organism-directed therapy when Aspergillus was identified, and mandibular disease could be managed with biopsy and close follow-up when aggressive surgery carried functional risk [12,13,17,18,21,29].

Taken together, these patterns support an integrated clinical-radiologic-pathologic diagnostic framework with early tissue confirmation rather than reliance on imaging alone for therapeutic decision-making (Table 5). When a patient is clinically stable and there is no immediate need for decompression or stabilization, imaging should be used to define lesion extent, assess structural risk, and guide biopsy planning before definitive oncologic or reconstructive intervention. This approach may prevent unnecessary wide oncologic resection in lesions that ultimately prove inflammatory, while still allowing urgent surgery when neurologic compromise, pathologic fracture, joint destruction, or extensive cortical instability is present [13,22,30,31]. In practice, this pathway requires coordination among orthopedic surgery, musculoskeletal radiology, pathology, and infectious disease to ensure adequate specimen handling for histology, culture, and, when needed, molecular or special-stain evaluation.

**Table 5:** Integrated clinical-radiologic-pathologic diagnostic and management framework for suspected XO XO, xanthogranulomatous osteomyelitis; TB, tuberculosis; FDG, fluorodeoxyglucose

Step	Decision point	Application
Step 1	Recognize aggressive imaging	A lytic/expansile lesion, cortical destruction, periosteal reaction, marrow edema, soft-tissue mass, or FDG uptake should trigger a malignancy workup.
Step 2	Avoid imaging-only diagnosis	XO, sarcoma, metastasis, TB, abscess, Langerhans cell histiocytosis, and Erdheim-Chester disease can overlap radiographically.
Step 3	Obtain tissue	Biopsy should include histopathology, bacterial culture, fungal culture, mycobacterial studies when relevant, and immunohistochemistry if histiocytic disease is considered.
Step 4	Match treatment to biology and biomechanics	Culture-positive cases may need targeted antimicrobials; unstable, destructive, spinal, or joint-involving lesions may require surgery.
Step 5	Report follow-up consistently	Future reports should include recurrence, imaging resolution, inflammatory markers, antibiotic duration, and functional outcome.

The present review also highlights a reporting gap that limits the ability to draw stronger conclusions. Follow-up duration, recurrence status, antibiotic selection, culture source, organism susceptibility, immune status, and functional outcomes were inconsistently described. This weakens the ability to compare antibiotics alone versus surgery, curettage versus resection, or observation versus reconstruction. Future case reports and case series should report a minimum dataset: lesion site and compartment; imaging features that triggered concern for malignancy; biopsy approach; histopathologic findings; culture method and source; antimicrobial regimen and duration; procedure performed; weight-bearing or functional status; complications; recurrence; and follow-up imaging. Standardized reporting would allow future reviews to move beyond descriptive synthesis toward more meaningful management recommendations.

Limitations

This review is limited by the rarity of XO and the case report/case series nature of the available evidence. Publication bias, inconsistent terminology, heterogeneous imaging descriptions, incomplete microbiologic detail, and variable follow-up constrain causal inference. The descriptive percentages reported in this manuscript were recalculated after manual verification and should be interpreted as study-level signals within a rare-literature dataset rather than formal incidence estimates. Small sample size, incomplete reporting, and the absence of comparative cohorts also preclude meta-analysis.

## Conclusions

XO remains an uncommon but clinically important mimic of malignant bone disease. In the published literature, destructive lytic imaging, cortical breach, periosteal reaction, soft-tissue abnormalities, and FDG avidity frequently prompted concern for malignancy before biopsy. Histopathologic confirmation with concurrent microbiologic evaluation provides the safest pathway to diagnosis, allowing clinicians to avoid inappropriate oncologic treatment while still addressing infection, fracture risk, neurologic compromise, or site-specific mechanical instability. Future reports should standardize microbiology, antimicrobial duration, functional recovery, recurrence, and follow-up imaging to enable more evidence-based management recommendations.
